# Supplementary material for: Extensive genomic diversity in Desulfovibrio reveals species-specific functional traits associated with disease
Source: Nat Commun. 2026 Oct 10;17:10655. doi: 10.1038/s41467-026-78314-1 (PMC13653922; doi:10.1038/s41467-026-78314-1)
Supplement: Supplementary file 2 [file 41467_2026_78314_MOESM2_ESM.pdf]

File name: Supplementary Data 1
Description: Statistics and host information for *Desulfovibrio* genome assemblies.

File name: Supplementary Data 2
Description: Metagenomic datasets used for new *Desulfovibrio* MAG identification.

File name: Supplementary Data 3
Description: *Desulfovibrio* prevalence in cohorts used for de novo MAG discovery.

File name: Supplementary Data 4
Description: Genomes reassigned to other genera within the Desulfovibrionaceae family using GTDB-tk.

File name: Supplementary Data 5
Description: Detailed information on the origin, availability and assembly statistics for the 24 human *Desulfovibrio* isolates.

File name: Supplementary Data 6
Description: Overview of all studies used to compile the *Desulfovibrio* database.

File name: Supplementary Data 7
Description: *Desulfovibrio sp900319575* genes that were enriched in the Netherlands and East Asia respectively. Two-sided Fisher's exact test was used to calculate the p-values. The adjusted p-values were obtained using the Benjamini-Hochberg (BH) method for multiple-testing correction.

File name: Supplementary Data 8
Description: Clusters of Orthologous Genes (COGs) significantly enriched in Crohn's disease (CD) vs healthy individuals (two-sided Fisher's exact test, adjusted p-value <0.25). The adjusted p-values were obtained using the Benjamini-Hochberg (BH) method for multiple-testing correction.

File name: Supplementary Data 9
Description: Clusters of Orthologous Genes (COGs) significantly enriched in colorectal cancer (CRC) vs healthy individuals (two-sided Fisher's exact test, adjusted p-value <0.25). The adjusted p-values were obtained using the Benjamini-Hochberg (BH) method for multiple-testing correction.

File name: Supplementary Data 10
Description: Hmm files for the curated microbial sulfur metabolism gene database.

File name: Supplementary Data 11
Description: MAGs identified in a deeply sequenced IBD cohort (FedericiS_2022).

File name: Supplementary Data 12
Description: The sulfur metabolism gene present in MAGs identified in a deeply sequenced IBD cohort (FedericiS_2022). In the matrix, "1" means "presence" and "0" means "absence".
