# Supplementary material for: Extensive genomic diversity in Desulfovibrio reveals species-specific functional traits associated with disease
Source: Nat Commun. 2026 Oct 10;17:10655. doi: 10.1038/s41467-026-78314-1 (PMC13653922; doi:10.1038/s41467-026-78314-1)
Supplement: Supplementary file 3 [file 41467_2026_78314_MOESM4_ESM.pdf]

nature portfolio

Corresponding author(s): Melanie Schirmer

Last updated by author(s): Sep 12, 2026

Reporting Summary

Nature Portfolio wishes to improve the reproducibility of the work that we publish. This form provides structure for consistency and transparency in reporting. For further information on Nature Portfolio policies, see our Editorial Policies and the Editorial Policy Checklist.

Statistics

For all statistical analyses, confirm that the following items are present in the figure legend, table legend, main text, or Methods section.

n/a	Confirmed	
☐	☒	The exact sample size (*n*) for each experimental group/condition, given as a discrete number and unit of measurement
☐	☒	A statement on whether measurements were taken from distinct samples or whether the same sample was measured repeatedly
☐	☒	The statistical test(s) used AND whether they are one- or two-sided<br>*Only common tests should be described solely by name; describe more complex techniques in the Methods section.*
☐	☒	A description of all covariates tested
☐	☒	A description of any assumptions or corrections, such as tests of normality and adjustment for multiple comparisons
☐	☒	A full description of the statistical parameters including central tendency (e.g. means) or other basic estimates (e.g. regression coefficient) AND variation (e.g. standard deviation) or associated estimates of uncertainty (e.g. confidence intervals)
☐	☒	For null hypothesis testing, the test statistic (e.g. *F*, *t*, *r*) with confidence intervals, effect sizes, degrees of freedom and *P* value noted<br>*Give P values as exact values whenever suitable.*
☒	☐	For Bayesian analysis, information on the choice of priors and Markov chain Monte Carlo settings
☒	☐	For hierarchical and complex designs, identification of the appropriate level for tests and full reporting of outcomes
☒	☐	Estimates of effect sizes (e.g. Cohen's *d*, Pearson's *r*), indicating how they were calculated

Our web collection on statistics for biologists contains articles on many of the points above.

Software and code

Policy information about availability of computer code

Data collection	No software was used for data collection
Data analysis	All the tools used for our analysis are publicly available, including: Trim Galore (v0.6.7), Porechop (https://github.com/rrwick/Porechop/), NanoFilt (v2.8.0), Unicycler (vv0.4.9), Kneaddata (https://github.com/biobakery/kneaddata), Megahit (v1.2.9), Maxbin 2, MetaBAT 2, CONCOCT (v1.1.0), metaWRAP (v1.3.2), bowtie2 (v2.4.2), GTDB-tk (v2.1.1), Mashtree (v1.2.0), GraPhlAn (v1.1.3), pyani (v0.2.12), prokka (v1.14.6),EggNOG-mapper (v2.1.64), InterProScan (v5.59-91.0), ABRicate (https://github.com/tseemann/abricate), Roary (http://sanger-pathogens.github.io/Roary/), FastTree (v2.1.11), R package Vegan, hmmscan (v3.3.2), cd-hit-est (v4.8.1), checkM (v1.1.3). Further details are specified in the methods section. Our custome code: https://github.com/schirmer-lab/Desulfovibrio_genomicAnalysis

For manuscripts utilizing custom algorithms or software that are central to the research but not yet described in published literature, software must be made available to editors and reviewers. We strongly encourage code deposition in a community repository (e.g. GitHub). See the Nature Portfolio guidelines for submitting code & software for further information.

Data

Policy information about availability of data

All manuscripts must include a data availability statement. This statement should provide the following information, where applicable:

- Accession codes, unique identifiers, or web links for publicly available datasets
- A description of any restrictions on data availability
- For clinical datasets or third party data, please ensure that the statement adheres to our policy

The Desulfovibrio genome database, including newly sequenced isolate assemblies and identified MAGs, are available at Zenodo: https://doi.org/10.5281/zenodo.17927643. The de novo reconstructed MAGs and isolate assemblies are also deposited in the European Nucleotide Archive (ENA) database under BioProject PRJEB123827. The MAGs from the Dutch cohorts (including DAG3 and Lifelines Next) were not included in the ENA submission because the corresponding metagenomic datasets are under restricted access. The corresponding metadata information is included in Supplementary Data 1. Detailed information for all isolates can be found in Supplementary Data 5. All newly isolated strains have been characterized and were deposited at the Weihenstephaner Stammsammlung at the Core Facility Microbiome, Technical University of Munich with WDCM number 1163 (https://ccinfo.wdcm.org/details?regnum=1163) under accession number WS 5745 - WS 5752 (further details in Supplementary Data 5). For strain MS028 biobank deposition was not possible due to ethics restriction for EEN patient samples. Two strains (BP2_2_G5_PP-3-Pallet_E04 and BP2_2_G7_PP-1-Pallet_F11) were isolated and are available through openBiome [98]. The description of the new species has been added to Supplementary information. Source Data are provided with this paper. All scripts used for the analysis are available at https://github.com/schirmer-lab/Desulfovibrio_genomicAnalysis and has been archived on Zenodo (DOI: 10.5281/zenodo.21997410). Other tools are documented in the methods section.

Research involving human participants, their data, or biological material

Policy information about studies with human participants or human data. See also policy information about sex, gender (identity/presentation), and sexual orientation and race, ethnicity and racism.

Reporting on sex and gender	To identify Desulfovibrio MAGs, we collected 11,017 metagenomic samples from 10 publicly cohort studies. Age and gender were described in the respective studies. The information about these studies can be found in Supplementary Table S2. We also isolated Desulfovibrio strains from patient samples with primary colorectal cancer and pediatric-onset IBD. The patient information is listed in Supplementary Table S5 with further information in the respective studies as indicated.
Reporting on race, ethnicity, or other socially relevant groupings	We collected 11,017 metagenomic samples from 10 publicly cohort studies expanding different health (e.g. healthy individuals, IBD, CRC and other diseases) and geographical areas, including China, Nepal, Africa, Netherlands, US, Germany, France and Israel. The phenotypic characteristics were described in their respective studies.
Population characteristics	The information about all studies can be found in Supplementary Table S2 and S6 with detailed phenotypic characteristics described in the respective study.
Recruitment	For Desulfovibrio isolations, samples from four cohorts were used. The recruitment information is available in the original publications for these cohorts.
Ethics oversight	Ethics information is specified in the methods. Specifically, we confirm that our research complies with all relevant ethical regulations. Bacterial isolations were performed on stool samples from a pediatric CD study (EEN study [94]) approved by the Ethics committee of the Ludwig-Maximilians University of Munich (approval No. 17-801; German Clinical Trials Register Accession No. DRKS00013306, date registration 19.03.2018) and a CRC cohort [95] (approval No. 375/16S) approved by the Ethics committee of the School of Medicine and Health TUM (2022-275-S-DFG-KK). Written consent was obtained from the participants of both studies, which includes broad microbial isolation efforts from patient samples, including Desulfovibrio spp..

Note that full information on the approval of the study protocol must also be provided in the manuscript.

Field-specific reporting

Please select the one below that is the best fit for your research. If you are not sure, read the appropriate sections before making your selection.

[x] Life sciences [ ] Behavioural & social sciences [ ] Ecological, evolutionary & environmental sciences

For a reference copy of the document with all sections, see nature.com/documents/nr-reporting-summary-flat.pdf

Life sciences study design

All studies must disclose on these points even when the disclosure is negative.

Sample size	We constructed a genomic database including 2,282 Desulfovibrio genomes. Specifically, we obtained 24 Desulfovibrio isolates from various human cohorts and public culture collections. We also downloaded genomes of 11 human isolates from NCBI RefSeq. Moreover, we identified 1,904 Desulfovibrio high-quality MAGs from 10,680 metagenomic samples from 9 human cohort studies with different health conditions. We also downloaded Desulfovibrio MAGs from NCBI RefSeq and other genomes reference databases.
Data exclusions	For strains from the same individual with an ANI value >99.9%, only one genome with the best assembly quality was kept. To remove duplicate entries between databases, if multiple genomes from the same original study were available with ANI values >99.5%, the genome with the highest completeness and lowest contamination was included in our downstream analysis.

Replication	Our bioinformatic analysis indicates that flagellated Desulfovibrio strains can elicit toll-like receptor 5 (TLR5) activation, which was experimentally confirmed. All experiments were performed in triplicates
Randomization	NA
Blinding	NA

Reporting for specific materials, systems and methods

We require information from authors about some types of materials, experimental systems and methods used in many studies. Here, indicate whether each material, system or method listed is relevant to your study. If you are not sure if a list item applies to your research, read the appropriate section before selecting a response.

Materials & experimental systems

n/a	Involved in the study	
☒	☐	Antibodies
☐	☒	Eukaryotic cell lines
☒	☐	Palaeontology and archaeology
☐	☒	Animals and other organisms
☒	☐	Clinical data
☒	☐	Dual use research of concern
☒	☐	Plants

Methods

n/a	Involved in the study	
☒	☐	ChIP-seq
☒	☐	Flow cytometry
☒	☐	MRI-based neuroimaging

Eukaryotic cell lines

Policy information about cell lines and Sex and Gender in Research

Cell line source(s)	HEK-Blue TLR5 reporter cell line (reference: hkb-htlr5, InvivoGen) reporting for murine TLR5 receptors and control cell line HEK-Blue Null1 without TLR gene overexpression (passages 7 to 9) were used to examine the activation of TLR5 by flagellated Desulfovibrio strains.
Authentication	Cell lines were commercially procured.
Mycoplasma contamination	All used cell lines (passages 1 and 2) were tested for mycoplasma contamination with the VenorGeM Mycoplasma Detection Kit (Art.-Nr.: 11-8025,Venor®GeM OneStep), PCR-based, and tested negatively.
Commonly misidentified lines (See ICLAC register)	None

Animals and other research organisms

Policy information about studies involving animals; ARRIVE guidelines recommended for reporting animal research, and Sex and Gender in Research

Laboratory animals	All mice were bred and maintained under either conventional or specific pathogen–free conditions at the School of Medicine, TUM, according to the guidelines of the Federation of Laboratory Animal Science Association. Mice were housed with a light:dark cycle of 12 hours, ambient temperature of 24°C, and humidity of 55%. At the end of the experiment, mice were euthanized, and small intestinal crypts were collected for generating organoids. All mice were euthanised with permission of the District Government of Upper Bavaria (License number 4-017-18).
Wild animals	*Provide details on animals observed in or captured in the field; report species and age where possible. Describe how animals were caught and transported and what happened to captive animals after the study (if killed, explain why and describe method; if released, say where and when) OR state that the study did not involve wild animals.*
Reporting on sex	sex was not taken into consideration.
Field-collected samples	*For laboratory work with field-collected samples, describe all relevant parameters such as housing, maintenance, temperature, photoperiod and end-of-experiment protocol OR state that the study did not involve samples collected from the field.*
Ethics oversight	No experiments were performed on live mice; therefore, no study protocol was required.

Note that full information on the approval of the study protocol must also be provided in the manuscript.

Plants

Seed stocks	NA
Novel plant genotypes	NA
Authentication	NA
