# Supplementary material for: Extensive genomic diversity in Desulfovibrio reveals species-specific functional traits associated with disease
Source: Nat Commun. 2026 Oct 10;17:10655. doi: 10.1038/s41467-026-78314-1 (PMC13653922; doi:10.1038/s41467-026-78314-1)
Supplement: Supplementary file 4 [file 41467_2026_78314_MOESM5_ESM.pdf]

nature portfolio

Peer Review File

Extensive genomic diversity in Desulfovibrio reveals species-specific functional traits associated with disease

Corresponding Author: Professor Melanie Schirmer

This manuscript has been previously reviewed at another journal. This document only contains information relating to versions considered at Nature Communications.

This file contains all reviewer reports in order by version, followed by all author rebuttals in order by version.

Version 0:

Reviewer comments:

Reviewer #1

(Remarks to the Author)
The authors have addressed most of my comments from the previous round and I believe the manuscript has improved a fair bit. I still want to highlight the fact that it is important to assess in some way the assembly error rate present in the MAGs constructed here and how that potentially impacts the identification of clusters at various ANIs.

Reviewer #2

(Remarks to the Author)
The authors have modified the manuscript in response to the remarks from Reviewer #2.

They have indicated that all the individual strains are available and from which organization they can be acquired. The database was also made accessible to the reviewers and will be open after publication.
Information about taurine metabolism pathway and its differential conservation in Desulfovibrio genomes were added in the main text (L. 394-404) and also in the context of diseases (L. 438-446). Three new figures related to taurine metabolism (14, 15 and 16) were added as Supplemental figures.
With the addition of a result section describing the exposure of wild-type and TLR5 knockout murine small intestinal organoids to recombinant flagelins from Desulfovibrio species, the authors provide further evidence of a capacity to modulate the innate immune response. That said, it may be worth adding a comment about the choice of small intestine versus colon organoids as Desulfovibrio species are often reported in the literature to be present mostly in the human colon.
The authors provided clarification in the text about the possible contribution of the assimilatory sulfate reduction pathway to the production in the gut (L.423-430.
They also removed the statement of geographic-specific increase of Desulfovibrio prevalence in the Dutch population and extended their samples database.
The naming and description of the novel Desulfovibrio isolates were added as Supplementary information as requested.

Other comments:

A comment about the inflammation-related production of tetrathionate in the gut and its metabolism leading to H2S production potentially further amplifying the inflammation.

L. 502: the urea uptake genes are distinct from the nitrogenase genes.

Overall, the authors provided satisfactory responses to Reviewer #2 comments. They strengthened the manuscript by expanding their database and analyses, enhancing their arguments and adding more experimental evidence.

Reviewer #3

(Remarks to the Author)
I would like to commend the authors for incorporating additional experiments using organoids to assess the role of Desulfovibrio flagellins in Toll-like receptor 5 activation. This significantly strengthens the manuscript and provides valuable functional support for the genomic observations.
However, several aspects of the revised manuscript raise concerns, particularly regarding interpretation of metabolic pathways and overstatement of functional relevance.

Major Comments
• The new analyses related to taurine metabolism by Desulfovibrio are difficult to reconcile with existing knowledge. The manuscript states that the tpa gene is “conserved” among Desulfovibrio; however, this contradicts previous studies. Notably, the cited literature appears to be used to support this statement, despite including conflicting information—even within the abstract of reference (1) (L397). This inconsistency needs to be resolved and the interpretation corrected.
• The manuscript classifies urease as a virulence factor; however, urease is a broadly distributed metabolic enzyme involved in nitrogen metabolism and environmental adaptation (e.g. survival under acidic conditions). While it can contribute to pathogenicity in specific organisms, its presence alone does not indicate virulence. The authors should refine this interpretation and avoid generalizing urease as a virulence determinant.
• The manuscript places substantial emphasis on the genomic potential of Desulfovibrio to produce H_2S. However, dissimilatory sulfate reduction and associated H_2S production are defining and well-established metabolic features of this genus. As such, the presence and conservation of these pathways are expected and do not constitute novel insight. The authors should better distinguish between known core metabolism and genuinely novel findings, and avoid overstating the significance of H_2S production capacity in Desulfovibrio.

Specific Comments
• L70ff: This should refer to sulfur compounds, as elemental sulfur is not being released.
• L72: This should specify some sulfate-reducing bacteria (SRBs), as not all can utilize all listed sulfur compounds.
• L170: How can D. longus be described as a novel species (sp. nov.) if it was already described in 1992? Please clarify.
• L202ff and Fig. 2a: The text refers to clade III, but this clade is not clearly indicated in the figure. It is therefore unclear what the authors are referring to.
• L223ff: The evidence presented does not demonstrate that these pathways are involved in colonization or pathogenesis. This conclusion appears overstated and should be revised.
• L244ff: Only kanamycin and ceftazidime are mentioned for MIC (should be lowercase). However, kanamycin has limited or no activity under anaerobic conditions, and more appropriate analogues exist for such testing. This should be addressed.
• L303: This result is not significant and should not be interpreted as such.
• L411ff: The role of organosulfur metabolism in the gut microbiome has previously been investigated in greater detail by (2). This key study is not discussed and should be included.
• L418–419: Supplementary Table 11 does not appear to support the statement made here. Please clarify.
• L502: The urea uptake system is not equivalent to nitrogenases. This statement is incorrect and should be revised.
• L757: There are multiple versions of Postgate media. The specific formulation and composition used should be clearly described.

Additional Comments
• The ethics statement regarding isolation of bacteria from human stool samples is missing and must be included.

References
1. S. C. Peck et al., A glycyl radical enzyme enables hydrogen sulfide production by the human intestinal bacterium Bilophila wadsworthia. Proc Natl Acad Sci U S A (2019).
2. P. G. Wolf et al., Diversity and distribution of sulfur metabolic genes in the human gut microbiome and their association with colorectal cancer. Microbiome 10, 64 (2022).

Version 1:

Reviewer comments:

Reviewer #1

(Remarks to the Author)
Based on the author’s response, I believe they have missed the fact that my point was about assembly correctness (e.g. error rate in bases, indels, as well as larger assembly errors). This is of course not the same as assembly quality which is often assessed via completeness and contamination metrics. A genome could have high completeness and low contamination and yet have hundreds of assembly errors. These errors would then impact all downstream analyses including the determination of species-level clusters. My query and concerns therefore remain unanswered here, though the robustness analysis that the authors show does have some independent value. I would encourage the authors to evaluate correctness for their assemblies and also check what impact it has on the downstream analysis and results that they report.

Reviewer #2

(Remarks to the Author)

The authors have addressed all my previous comments in a satisfactory manner.

Reviewer #3

(Remarks to the Author)
I would like to thank the authors for their revisions and for addressing the majority of my previous comments. Most of my concerns have been satisfactorily resolved.
However, I remain unconvinced by the interpretation regarding the widespread conservation of tpa among Desulfovibrio spp. The authors argue that strong BLAST hits support the presence of tpa homologues, but this does not address my original concern. The study they cite did not only investigate protein expression — it also analysed genomic context and showed that the canonical taurine-utilisation cluster is absent from D. desulfuricans DSM642 and O. alaskensis G20. The presence of proteins with sequence similarity to Tpa therefore cannot, by itself, be taken as evidence that these organisms encode functional taurine:pyruvate aminotransferases.
To better understand this discrepancy, I independently inspected the genomes discussed by the authors. The reported D. desulfuricans DSM642 homologue (VBIDesDes264612_0555) is annotated as an N-acetylornithine aminotransferase and carries the PRK02627 acetylornithine aminotransferase domain. Moreover, while the characterised Tpa protein belongs to COG0166, this COG is not specific to taurine metabolism and includes aminotransferases involved in biotin biosynthesis as well. The widespread occurrence of proteins assigned to this COG may therefore reflect conservation of broadly distributed aminotransferases rather than conservation of taurine metabolism per se.
I would have expected the authors to carry out these analyses before concluding that tpa is conserved or widespread among Desulfovibrio spp. As it stands, I do not think the conclusion is sufficiently supported. If the authors wish to maintain this claim, they should provide direct evidence that isolates carrying the more divergent putative homologues can actually utilise taurine — for example by demonstrating growth on taurine and showing taurine consumption and/or production of the expected downstream metabolites. Otherwise, I would recommend describing these genes as putative aminotransferases or putative tpa homologues and moderating the corresponding conclusions accordingly.

Version 2:

Reviewer comments:

Reviewer #1

(Remarks to the Author)
Thank you for considering my suggestion and addressing it with a specific experiment. I would have liked to see results where large assembly errors were simulated as well. The acknowledged limitation in the discussion section partially addresses this.

Open Access This Peer Review File is licensed under a Creative Commons Attribution 4.0 International License, which permits use, sharing, adaptation, distribution and reproduction in any medium or format, as long as you give appropriate credit to the original author(s) and the source, provide a link to the Creative Commons license, and indicate if changes were

made.
In cases where reviewers are anonymous, credit should be given to 'Anonymous Referee' and the source.
The images or other third party material in this Peer Review File are included in the article's Creative Commons license, unless indicated otherwise in a credit line to the material. If material is not included in the article's Creative Commons license and your intended use is not permitted by statutory regulation or exceeds the permitted use, you will need to obtain permission directly from the copyright holder.
To view a copy of this license, visit https://creativecommons.org/licenses/by/4.0/

REVIEWER COMMENTS

Reviewer #1 (Remarks to the Author):

The authors have addressed most of my comments from the previous round and I believe the manuscript has improved a fair bit. I still want to highlight the fact that it is important to assess in some way the assembly error rate present in the MAGs constructed here and how that potentially impacts the identification of clusters at various ANIs.

We thank the reviewer for the positive feedback on our revised manuscript.

To address the reviewer's remaining concern about the potential influence of assembly errors on the identified *Desulfovibrio* species clusters, we evaluated the effects of different levels of assembly quality (estimated by completeness and contamination) and ANI cutoffs on the identification of new *Desulfovibrio* species cluster information (Fig. R1). When applying a more stringent ANI threshold (96%, 97%, 98% or 99%), 69 of the 70 genomes assigned to clade I remain in this cluster, indicating the robustness of this cluster. Furthermore, if we filter the genomes using a highly stringent completeness threshold (99%) and cluster them at 95% ANI, the largest cluster still consists exclusively of genomes from clade I, where approximately 50% of genomes from clade I are retained. Similarly, when we filter these uncharacterized genomes with a very low contamination threshold (1%), the largest cluster still consists of only genomes assigned to clade I, although the number of genomes decreased to 25 in this case. For clade II and clade III, each containing 3 genomes, the cluster assignment remains unchanged even if stricter criteria are applied (>97% ANI or >92% completeness or <3% contamination). Overall these results support the robustness and reliability of the novel species clusters identified in this study.

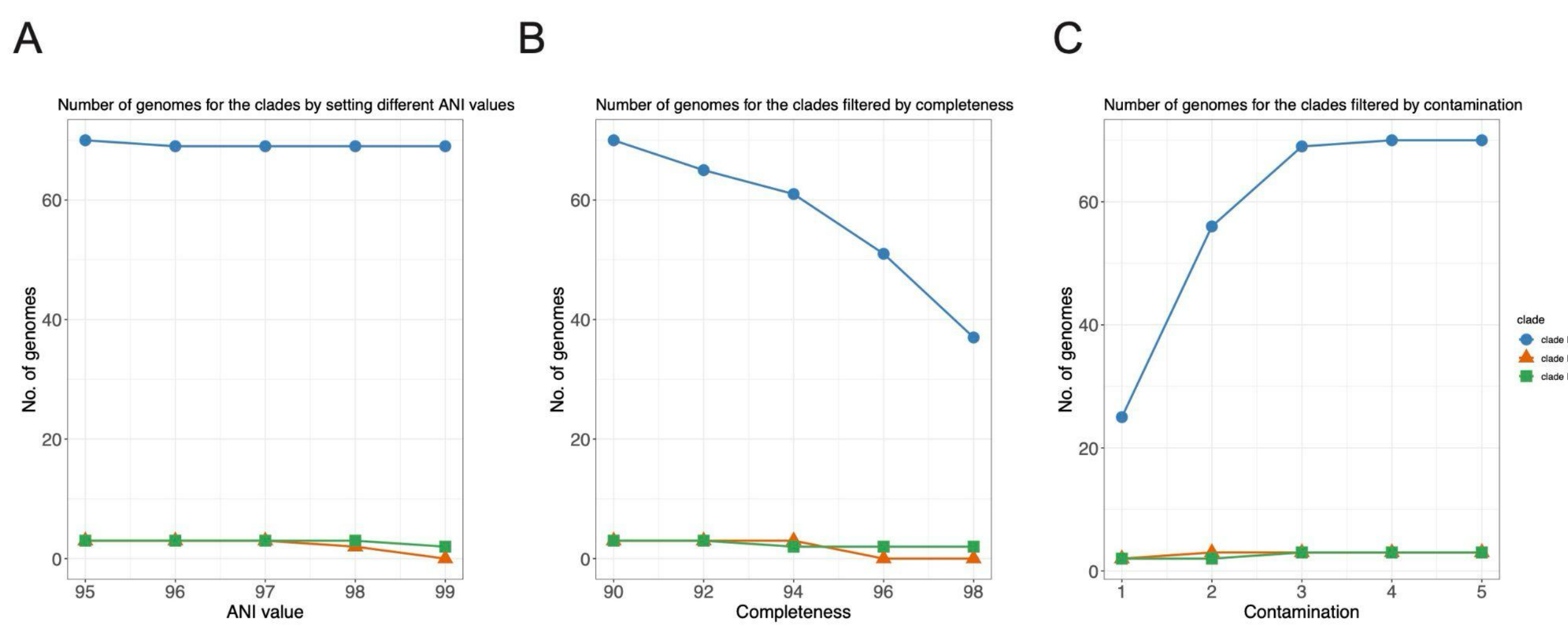

Fig. R1. The effect of ANI, completeness and contamination on *Desulfovibrio* species clusters.

Reviewer #2 (Remarks to the Author):

The authors have modified the manuscript in response to the remarks from Reviewer #2.

They have indicated that all the individual strains are available and from which organization they can be acquired. The database was also made accessible to the reviewers and will be open after publication.

Information about taurine metabolism pathway and its differential conservation in Desulfovibrio genomes were added in the main text (L. 394-404) and also in the context of diseases (L. 438-446). Three new figures related to taurine metabolism (14, 15 and 16) were added as Supplemental figures.

With the addition of a result section describing the exposure of wild-type and TLR5 knockout murine small intestinal organoids to recombinant flagelins from Desulfovibrio species, the authors provide further evidence of a capacity to modulate the innate immune response. That said, it may be worth adding a comment about the choice of small intestine versus colon organoids as Desulfovibrio species are often reported in the literature to be present mostly in the human colon.

The authors provided clarification in the text about the possible contribution of the assimilatory sulfate reduction pathway to the production in the gut (L.423-430. They also removed the statement of geographic-specific increase of Desulfovibrio prevalence in the Dutch population and extended their samples database. The naming and description of the novel Desulfovibrio isolates were added as Supplementary information as requested.

We thank the reviewer for the careful consideration of our revised manuscript.

While the bacterial burden is generally higher in the colon, small intestinal organoids are often chosen to investigate epithelial TLR5 signaling [1-3], as they exhibit a more complex epithelial spatial organization [4, 5]. For our study, we specifically focused on the TLR5-mediated immune response induced by purified *Desulfovibrio* flagellin and thus opted for small intestinal organoids. Moreover, *Desulfovibrio* has previously also been detected in the human small intestine [6, 7]. Thus investigating interactions between *Desulfovibrio* flagellins and TLR5 in the context of the small intestine remains biologically relevant. We now also added the following text to the revised manuscript:

> "We further profiled immune system interactions involving *Desulfovibrio* flagellins using murine small intestinal organoids (*Tlr5* knocked-out vs wild type organoids), which represent a well established model for the investigation of epithelial TLR5 signaling [1-3]."

Other comments:

A comment about the inflammation-related production of tetrathionate in the gut and its metabolism leading to H2S production potentially further amplifying the inflammation.

Following the reviewer's suggestion, we added a comment on this point to the revised manuscript:

> "However, genes involved in tetrathionate-related H_2S production were highly abundant in the IBD gut microbiome compared to healthy individuals and tetrathionate respiration has been shown to confer a growth advantage for pathogenic bacteria during inflammation [8]. Collectively, this suggests that inflammation-associated tetrathionate respiration may contribute to a positive feedback loop that exacerbates gut inflammation through microbial H_2S production."

L. 502: the urea uptake genes are distinct from the nitrogenase genes.

We thank the reviewer for pointing this out and have adjusted the language in the revised manuscript as follows:

> "Importantly, expression of *Desulfovibrio* nitrogenase genes in patient stool samples suggests that nitrogen fixation is active in the human gut [9]."

Overall, the authors provided satisfactory responses to Reviewer #2 comments. They strengthened the manuscript by expanding their database and analyses, enhancing their arguments and adding more experimental evidence.

Reviewer #3 (Remarks to the Author):

I would like to commend the authors for incorporating additional experiments using organoids to assess the role of Desulfovibrio flagellins in Toll-like receptor 5 activation. This significantly strengthens the manuscript and provides valuable functional support for the genomic observations. However, several aspects of the revised manuscript raise concerns, particularly regarding interpretation of metabolic pathways and overstatement of functional relevance.

Major Comments

The new analyses related to taurine metabolism by Desulfovibrio are difficult to reconcile with existing knowledge. The manuscript states that the tpa gene is "conserved" among Desulfovibrio; however, this contradicts previous studies. Notably, the cited literature appears to be used to support this statement, despite including conflicting information—even within the abstract of reference (1) (L397). This inconsistency needs to be resolved and the interpretation corrected.

We thank the reviewer for the positive feedback on our revised manuscript.

To address the reviewer's concerns regarding the contradiction in terms of taurine metabolism capacity of *Desulfovibrios*, we would like to emphasize that our investigation of genes involved in taurine metabolism is based on genomic analysis, whereas the referenced study [10] investigated protein expression of taurine metabolism in specific culture conditions. These complementary methods capture different aspects, where genomic analyses are inherently limited to the detection of the presence of genes and their genomic variation, but do not capture changes in transcript and protein expression in response to specific experimental conditions.

Our database includes the *Desulfovibrio desulfuricans* DSM642 genome that was used in the referenced study. It is also important to note that the second *Desulfovibrio* strain mentioned in the corresponding abstract, i.e. *Desulfovibrio alaskensis* G20, has since been reclassified as *Oleidesulfovibrio alaskensis* G20. We further re-examined the alignment of the *tpa* gene against this particular *D. desulfuricans* genome, which indicated a very strong match for *tpa* with an e-value of 1.8E-74 and provides evidence that this strain encodes a *tpa* homolog. As the presence of a particular gene does not necessarily indicate activity and bacteria can show differences in their metabolism in response to different culture conditions, we added the following point to the results section and also cite the respective study in the revised manuscript:

> "While many taurine metabolism genes (e.g. taurine pyruvate aminotransferase [*tpa*] and sulfoacetaldehyde acetyltrasferase [*xsc*]) were well conserved across *Desulfovibrio* spp. (**Supplementary Fig. 15**), *D. desulfuricans* DSM642 previously showed a lack of Tpa protein expression [10]. This highlights the importance of having culturable isolates available that can be tested in different culture conditions in combination with functional readouts to further investigate taurine metabolism in *Desulfovibrio*."

The manuscript classifies urease as a virulence factor; however, urease is a broadly distributed metabolic enzyme involved in nitrogen metabolism and environmental adaptation (e.g. survival under acidic conditions). While it can contribute to pathogenicity in specific organisms, its presence alone does not indicate virulence. The authors should refine this interpretation and avoid generalizing urease as a virulence determinant.

We thank the reviewer for pointing this out and rephrased the text as follows:

> "Genomic analyses showed extensive species diversity and revealed disease-associated functional traits, including flagellin, and adaptation-associated genes (i.e. ureases). [...]
>
> Urease is broadly involved in nitrogen metabolism and environmental adaptation, while it also contributes to the virulence in some pathogenic bacteria [11]. [...]
>
> Urease was associated with gut dysbiosis and is hypothesized to contribute to CD pathogenesis [12, 13]."

The manuscript places substantial emphasis on the genomic potential of Desulfovibrio to produce H_2S. However, dissimilatory sulfate reduction and associated H_2S production are defining and well-established metabolic features of this genus. As such, the presence and conservation of these pathways are expected and do not constitute novel insight. The authors should better distinguish between known core metabolism and genuinely novel findings, and avoid overstating the significance of H_2S production capacity in Desulfovibrio.

To avoid overstating the significance of H_2S production capacity via dissimilatory sulfate reduction of *Desulfovibrio* spp., we modify the statement as follows:

> "Overall, these findings confirm the established role of dissimilatory sulfate reduction as a conserved core metabolic feature across a wide range of *Desulfovibrio* species."

Moreover, we revised the section heading to "Comparison of sulfur metabolism capabilities across a wide range of *Desulfovibrio* spp." to more clearly emphasize that this analysis aimed to assess the distribution of sulfur metabolism across different *Desulfovibrio* species.

Specific Comments

- L70ff: This should refer to sulfur compounds, as elemental sulfur is not being released.

We thank the reviewer for pointing this out and we changed the text from the release of "sulfur" to the release of "sulfur compounds".

- L72: This should specify some sulfate-reducing bacteria (SRBs), as not all can utilize all listed sulfur compounds.

Following the reviewer's suggestion, we modified the statement as follows:

> "Furthermore, some sulfate-reducing gut bacteria, including *Desulfovibrio*, *Desulfobacter* and *Desulfobulbus,* reduce certain inorganic sulfur compounds, such as sulfite, sulfate, thiosulfate or tetrathionate, to H_2S [14]".

- L170: How can D. longus be described as a novel species (sp. nov.) if it was already described in 1992? Please clarify.

The species to which the reviewer refers has since been renamed as "*Paucidesulfovibrio longus*" [15, 16] and is thus not part of the *Desulfovibrio* genus anymore. However, to avoid any confusion, we changed the name for this species to *D. elongatum* and modified the text accordingly.

- L202ff and Fig. 2a: The text refers to clade III, but this clade is not clearly indicated in the figure. It is therefore unclear what the authors are referring to.

We apologize for the confusion and added an additional reference to Fig. 1d, which includes the information regarding clade III. Overall, this sentence summarises that flagellin genes were detected in specific *Desulfovibrio* species, including the newly identified clade III.

- L223ff: The evidence presented does not demonstrate that these pathways are involved in colonization or pathogenesis. This conclusion appears overstated and should be revised.

We thank the reviewer for raising this concern and have rephrased this sentence as follows:

> "These results indicate that *Desulfovibrio* species encode different metabolic pathways related to energy transfer involving sulfate respiration and functional traits (i.e. *ure*) that have been shown to be involved in host-associated adaptation in pathogenic bacteria."

- L244ff: Only kanamycin and ceftazidime are mentioned for MIC (should be lowercase). However, kanamycin has limited or no activity under anaerobic conditions, and more appropriate analogues exist for such testing. This should be addressed.

While we agree with the reviewer that kanamycin has limited clinical relevance for anaerobic bacteria, we choose this antibiotic for this initial characterisation as it is widely used in *in vitro* studies, including for anaerobic bacteria [17]. Our goal here was to investigate differences in antibiotic resistance between *Desulfovibrio* strains containing different panels of antibiotic resistance genes. To this end, we used common antibiotics in connection with the identified genes (chloramphenicol / beta-lactams => ampicillin, ceftazidime) as well as additional ones, including

tetracycline and kanamycin. With this, we were able to show that the antibiotic resistance pattern was quite varied, independently of the presence of resistance genes. We also saw some activity of kanamycin against specific *Desulfovibrio* strains. Even if kanamycin has limited relevance as a clinical antibiotic, we believe that the 5 chosen antibiotics support the presented conclusion. A more thorough investigation of the molecular characteristics of these human isolates (including clinical antibiotic profiling) will be conducted as part of a future publication focussing entirely on describing these new species. For transparency, we added this current limitation to the future outlook section:

> "Lastly, our characterization of the isolates from three new putative *Desulfovibrio* species will require additional investigations to fully identify their molecular characteristics, including testing clinically relevant antibiotics."

Furthermore, all occurrences of kanamycin and ceftazidime were changed to lowercase lettering.

- L303: This result is not significant and should not be interpreted as such.

We apologize for the confusion and would like to clarify that this value represents an adjusted p-value (adj. $p=0.067$ for *hipA* comparing the prevalence in UC vs. healthy). In our analysis we applied a threshold of adjusted p-value < 0.25 to identify disease-associated functional traits, as it is common for microbiome studies [18-20]. Thus the enrichment of toxin *hipA* in UC meets our criteria for significance. For further clarity, we added the method used for multiple testing correction to the respective sentence.

- L411ff: The role of organosulfur metabolism in the gut microbiome has previously been investigated in greater detail by (2). This key study is not discussed and should be included.

We thank the reviewer for pointing this out and added the respective citation to the revised manuscript:

> "These results indicate an increase in bacteria encoding H_2S-associated taurine and cysteine degradation pathways in IBD patients, which is in line with previously reported associations between organic sulfur-metabolizing bacteria and CRC risk [21]. Collectively, this supports the hypothesis that organic sulfur metabolism may represent an important H_2S source in the disease-associated microbiome."

- L418–419: Supplementary Table 11 does not appear to support the statement made here. Please clarify.

To improve this part, we added an additional table (Supplementary Table 12) that contains information on the presence of sulfur metabolism genes in all MAGs identified in the respective IBD cohort, including genes involved in dissimilatory sulfate reduction. The corresponding sentence was modified as follows:

> "Dissimilatory sulfate reduction was encoded by 40 of 54 (74.07%) *Desulfovibrio* MAGs spanning 7 known and one uncharacterized species (**Supplementary Table 11+12**)."

- L502: The urea uptake system is not equivalent to nitrogenases. This statement is incorrect and should be revised.

We thank the reviewer for pointing this out and have corrected the statement:

> "Importantly, expression of *Desulfovibrio* nitrogenase genes in patient stool samples suggests that nitrogen fixation is active in the human gut [9]."

- L757: There are multiple versions of Postgate media. The specific formulation and composition used should be clearly described.

We apologize for the confusion. We used two different types of Postgate media: modified Postgate-lactate medium and modified Postgate-pyruvate medium. To further clarify this, we have changed the text accordingly (using "modified Postgate-lactate medium" and "modified Postgate-pyruvate medium") and added the specific media composition to the methods section:

> "For this, 1 g of frozen fecal material (20 % glycerol) was transferred into 10 ml of anaerobe modified Postgate-lactate medium (0.5 g K_2HPO_4, 1.0 g NH_4Cl, 1.0 g Na_2SO_4, 0.1 g $CaCl_2$ x 2 H_2O, 2.0 g $MgSO_4$ x 7 H_2O, 2.0 g Na-DL-lactate, 1.0 g Yeast extract, 0.5 ml Na-resazurin (0.1 % w/v), 0.5 g $FeSo_4$ x 7 H_2O, 0.1 g Na-thioglycolate, 0.1 g Ascorbic acid) in Hungate tubes previously sparged with N_2. […]"

> "Single colonies were incubated in 10 ml modified Postgate-pyruvate medium (0.5g K_2HPO_4, 1.0g NH_4Cl, 1.0g Na_2SO_4, 0.1g $CaCl_2$ x 2 H_2O, 2.0g $MgSO_4$ x 7 H_2O, 5.0g Sodium pyruvate, 1.0g Yeast extract, 0.5ml Na-resazurin (0.1% w/v), 0.1g Na-thioglycolate, 0.1g Ascorbic acid) for 48 hours in bacterial tubes."

Additional Comments

- The ethics statement regarding isolation of bacteria from human stool samples is missing and must be included.

We added the following information about the bacterial strain isolation to the ethic statement of the revised manuscript:

> "Further *Desulfovibrio* isolates were obtained from stool samples from the Lifelines Dutch microbiome cohort and the 1000IBD cohort (University Medical Center Groningen Medical Ethical Committee: IRB numbers 2017.152, 2016/424 [GE-ID], 2014/291 [Rise-Up]) and two healthy US donors from OpenBiome (ethics approval MIT - COUHES protocol #1612797956). Ethic approvals for each cohort allowed for collecting stool samples in glycerol and subsequent isolation of live bacteria."
>
> "Furthermore, we used stool samples from a pediatric CD study (EEN study [22]) approved by the Ethics committee of the Ludwig-Maximilians University of Munich (approval No. 17-801; German Clinical Trials Register Accession No. DRKS00013306, date registration 19.03.2018) and a CRC cohort described in [23] (approval No. 375/16S) approved by the Ethics committee of the School of Medicine and Health, TUM (2022-275-S-DFG-KK), both of which permit bacterial isolation from patient samples."

References

1. S. C. Peck et al., A glycyl radical enzyme enables hydrogen sulfide production by the human intestinal bacterium Bilophila wadsworthia. Proc Natl Acad Sci U S A (2019).
2. P. G. Wolf et al., Diversity and distribution of sulfur metabolic genes in the human gut microbiome and their association with colorectal cancer. Microbiome 10, 64 (2022).

References

1. Wahida, A., et al., *XIAP restrains TNF-driven intestinal inflammation and dysbiosis by promoting innate immune responses of Paneth and dendritic cells.* Sci Immunol, 2021. **6**(65): p. eabf7235.
2. Clasen, S.J., et al., *Silent recognition of flagellins from human gut commensal bacteria by Toll-like receptor 5.* Sci Immunol, 2023. **8**(79): p. eabq7001.
3. Price, A.E., et al., *A Map of Toll-like Receptor Expression in the Intestinal Epithelium Reveals Distinct Spatial, Cell Type-Specific, and Temporal Patterns.* Immunity, 2018. **49**(3): p. 560-575 e6.
4. Daghero, H., et al., *Jejunum-derived NF-kappaB reporter organoids as 3D models for the study of TNF-alpha-induced inflammation.* Sci Rep, 2022. **12**(1): p. 14425.
5. Rahmani, S., et al., *Intestinal organoids: A new paradigm for engineering intestinal epithelium in vitro.* Biomaterials, 2019. **194**: p. 195-214.
6. Leite, G.G.S., et al., *Mapping the Segmental Microbiomes in the Human Small Bowel in Comparison with Stool: A REIMAGINE Study.* Dig Dis Sci, 2020. **65**(9): p. 2595-2604.
7. Singh, S.B., A. Carroll-Portillo, and H.C. Lin, *Desulfovibrio in the Gut: The Enemy within?* Microorganisms, 2023. **11**(7).
8. Winter, S.E., et al., *Gut inflammation provides a respiratory electron acceptor for Salmonella.* Nature, 2010. **467**(7314): p. 426-9.
9. Sayavedra, L., et al., *Desulfovibrio diazotrophicus sp. nov., a sulfate-reducing bacterium from the human gut capable of nitrogen fixation.* Environ Microbiol, 2021. **23**(6): p. 3164-3181.
10. Peck, S.C., et al., *A glycyl radical enzyme enables hydrogen sulfide production by the human intestinal bacterium Bilophila wadsworthia.* Proc Natl Acad Sci U S A, 2019. **116**(8): p. 3171-3176.
11. Rutherford, J.C., *The emerging role of urease as a general microbial virulence factor.* PLoS Pathog, 2014. **10**(5): p. e1004062.
12. Bury-Mone, S., et al., *The Helicobacter pylori UreI protein: role in adaptation to acidity and identification of residues essential for its activity and for acid activation.* Mol Microbiol, 2001. **42**(4): p. 1021-34.
13. Ni, J., et al., *A role for bacterial urease in gut dysbiosis and Crohn's disease.* Sci Transl Med, 2017. **9**(416).
14. Blachier, F., et al., *Production of hydrogen sulfide by the intestinal microbiota and epithelial cells and consequences for the colonic and rectal mucosa.* Am J Physiol Gastrointest Liver Physiol, 2021. **320**(2): p. G125-G135.
15. Magot, M., et al., *Desulfovibrio longus sp. nov., a sulfate-reducing bacterium isolated from an oil-producing well.* Int J Syst Bacteriol, 1992. **42**(3): p. 398-403.
16. Waite, D.W., et al., *Proposal to reclassify the proteobacterial classes Deltaproteobacteria and Oligoflexia, and the phylum Thermodesulfobacteria into four phyla reflecting major functional capabilities.* Int J Syst Evol Microbiol, 2020. **70**(11): p. 5972-6016.
17. Gajdacs, M., G. Spengler, and E. Urban, *Identification and Antimicrobial Susceptibility Testing of Anaerobic Bacteria: Rubik's Cube of Clinical Microbiology?* Antibiotics (Basel), 2017. **6**(4).
18. Jacobs, J.P., et al., *Multi-omics profiles of the intestinal microbiome in irritable bowel syndrome and its bowel habit subtypes.* Microbiome, 2023. **11**(1): p. 5.

19. Beck, L.C., et al., *Strain-specific impacts of probiotics are a significant driver of gut microbiome development in very preterm infants.* Nat Microbiol, 2022. **7**(10): p. 1525-1535.
20. Kaelin, E.A., et al., *Longitudinal gut virome analysis identifies specific viral signatures that precede necrotizing enterocolitis onset in preterm infants.* Nat Microbiol, 2022. **7**(5): p. 653-662.
21. Wolf, P.G., et al., *Diversity and distribution of sulfur metabolic genes in the human gut microbiome and their association with colorectal cancer.* Microbiome, 2022. **10**(1): p. 64.
22. Hacker, D., et al., *Exclusive enteral nutrition initiates individual protective microbiome changes to induce remission in pediatric Crohn's disease.* Cell Host Microbe, 2024.
23. Ecker, J., et al., *The Colorectal Cancer Lipidome: Identification of a Robust Tumor-Specific Lipid Species Signature.* Gastroenterology, 2021. **161**(3): p. 910-923 e19.

REVIEWER COMMENTS

Reviewer #1 (Remarks to the Author):

Based on the author's response, I believe they have missed the fact that my point was about assembly correctness (e.g. error rate in bases, indels, as well as larger assembly errors). This is of course not the same as assembly quality which is often assessed via completeness and contamination metrics. A genome could have high completeness and low contamination and yet have hundreds of assembly errors. These errors would then impact all downstream analyses including the determination of species-level clusters. My query and concerns therefore remain unanswered here, though the robustness analysis that the authors show does have some independent value. I would encourage the authors to evaluate correctness for their assemblies and also check what impact it has on the downstream analysis and results that they report.

We revisited the comments from Reviewer 1 also from the previous rounds of revisions to address this last remaining concern. As the reviewer highlighted in particular insertions, deletions and base errors and previously specifically inquired about the impact of assembly errors on the novel cluster identifications, we focused on this first.

We randomly introduced insertion, deletion and substitution errors into the genomes contained in novel cluster I, II and III mimicking different error rates. Error rates in Illumina sequencing are generally low and estimated to be around 0.1% (NovaSeq 6000 [1]). For the assemblies, these error rates should be substantially lower, as assemblers (including MEGAHIT [2]) commonly iterate through a range of k-mer values in a stepwise fashion and correct sequencing errors at each iteration to reduce the impact of sequencing errors.

For our analysis, we chose a range of error rates at 0.01 %, 0.1 %, 0.5%, 1%. We were able to show that even in the case of these high error rates the new species clusters I, II and III remain robust (Table R1). More specifically, only one genome assignment to cluster I changed even at error rates of 0.5% and 1%, while 69 of the 70 genomes assigned to clade I remained in this cluster and the cluster assignment for clade II and clade III remained unchanged.

Table R1. Number of genomes in the new species clusters across varying error rates.

Error types	Error rate	clade I	clade II	clade III
deletion	0.01%	70	3	3
deletion	0.10%	70	3	3
deletion	0.50%	69	3	3
deletion	1%	69	3	3
insertion	0.01%	70	3	3
insertion	0.10%	70	3	3
insertion	0.50%	69	3	3

insertion	1%	69	3	3
substitution	0.01%	70	3	3
substitution	0.10%	70	3	3
substitution	0.50%	69	3	3
substitution	1%	69	3	3

In addition to the species-level clustering, other types of analyses used for our study include the identification of orthologous groups (COGs) within species clusters, as well as antibiotic resistance genes, virulence factors and sulfur metabolism genes. All of these analyses focus on the species level and would not be affected by these types of errors within individual genomes of the cluster.

Furthermore, larger assembly errors can result from repeats within a genome, including repeat collapses, insertions, deletions and inversions and were previously described as the primary types of assembly errors [3]. Estimates for these types of errors either require a high-quality known reference genome as the ground truth or rely on features of the assembled data itself, seeking to identify internal inconsistencies indicative of potential assembly errors for de novo identification. The purpose of our study was to address the lack of reference genomes for *Desulfovibrio* spp., a species that remains difficult to isolate and culture and thus lacks reference genomes for most species. Further, the de novo methods for the evaluation of assembly errors often show high false positive rates and distinguishing mis-assemblies and true sequence differences remains challenging [3]. Nevertheless, the main effects of these types of large assembly errors on our analysis would involve genes missing due to assembly errors rather than these genes being truly absent in the respective genome. As the majority of our gene analyses was conducted at the species level and species-specific signals (e.g. COGs, virulence factors and ARGs, which were shown in Fig. 2) were always present/absent in over 95% of the genomes of the corresponding species, this should make these results robust against mis-assemblies of individual genomes within a species.

In summary, our analysis represents bioinformatic state-of-the-art approaches for studying bacteria that remain challenging to culture. As we cannot rule out that assembly errors are present in the reconstructed reference genomes of our database and the genomes obtained from other databases, we added this to the limitation section of the revised manuscript:

> “While we used state- of- the- art methods to construct MAGs and applied strict completeness and contamination filters to all genomes, we cannot exclude the presence of potential assembly errors within genomes in our database.”

Reviewer #2 (Remarks to the Author):

The authors have addressed all my previous comments in a satisfactory manner.

Reviewer #3 (Remarks to the Author):

I would like to thank the authors for their revisions and for addressing the majority of my previous comments. Most of my concerns have been satisfactorily resolved.

However, I remain unconvinced by the interpretation regarding the widespread conservation of tpa among Desulfovibrio spp. The authors argue that strong BLAST hits support the presence of tpa homologues, but this does not address my original concern. The study they cite did not only investigate protein expression — it also analysed genomic context and showed that the canonical taurine-utilisation cluster is absent from D. desulfuricans DSM642 and O. alaskensis G20. The presence of proteins with sequence similarity to Tpa therefore cannot, by itself, be taken as evidence that these organisms encode functional taurine:pyruvate aminotransferases.

To better understand this discrepancy, I independently inspected the genomes discussed by the authors. The reported D. desulfuricans DSM642 homologue (VBIDesDes264612_0555) is annotated as an N-acetylornithine aminotransferase and carries the PRK02627 acetylornithine aminotransferase domain. Moreover, while the characterised Tpa protein belongs to COG0166, this COG is not specific to taurine metabolism and includes aminotransferases involved in biotin biosynthesis as well. The widespread occurrence of proteins assigned to this COG may therefore reflect conservation of broadly distributed aminotransferases rather than conservation of taurine metabolism per se.

I would have expected the authors to carry out these analyses before concluding that tpa is conserved or widespread among Desulfovibrio spp. As it stands, I do not think the conclusion is sufficiently supported. If the authors wish to maintain this claim, they should provide direct evidence that isolates carrying the more divergent putative homologues can actually utilise taurine — for example by demonstrating growth on taurine and showing taurine consumption and/or production of the expected downstream metabolites. Otherwise, I would recommend describing these genes as putative aminotransferases or putative tpa homologues and moderating the corresponding conclusions accordingly.

We thank the reviewer for this additional input. We have modified the respective sentence referring to *putative tpa* homologues in the revised manuscript and clarified that these genes signals were detected in *Desulfovibrio* spp. based on sequence similarity. We agree with the reviewer that the detection of a gene based on sequence similarity does not necessarily mean that the gene is functional. Therefore, we also mention that *D. desulfuricans* DSM642 previously showed a lack of Tpa protein expression and highlight the importance of having culturable isolates available for experimental follow-up investigations of computationally predicted signals. We did not mention *Oleidesulfovibrio alaskensis* G20 as it is no longer classified as a *Desulfovibrio* spp. and is thus not included in our analysis.

“Many taurine metabolism genes (e.g. putative taurine pyruvate aminotransferase [tpa] and sulfoacetaldehyde acetyltrasferase [xsc]) were detected in *Desulfovibrio* spp. (based on sequence similarity, **Supplementary Fig. 15**). However*, D. desulfuricans* DSM642 previously showed a lack of Tpa protein expression [4], highlighting the importance of having culturable isolates that can be tested in different culture conditions in combination with functional readouts to further investigate taurine metabolism signals in *Desulfovibrio*.”

References

1. Stoler, N. and A. Nekrutenko, *Sequencing error profiles of Illumina sequencing instruments.* NAR Genom Bioinform, 2021. **3**(1): p. lqab019.
2. Li, D., et al., *MEGAHIT v1.0: A fast and scalable metagenome assembler driven by advanced methodologies and community practices.* Methods, 2016. **102**: p. 3-11.
3. Olson, N.D., et al., *Metagenomic assembly through the lens of validation: recent advances in assessing and improving the quality of genomes assembled from metagenomes.* Brief Bioinform, 2019. **20**(4): p. 1140-1150.
4. Peck, S.C., et al., *A glycyl radical enzyme enables hydrogen sulfide production by the human intestinal bacterium Bilophila wadsworthia.* Proc Natl Acad Sci U S A, 2019. **116**(8): p. 3171-3176.
